# Assessment of low dietary inclusion of nutraceuticals derived from microalgae to enhance intestinal function in gilthead seabream (*Sparus aurata*) juveniles

**DOI:** 10.1038/s41598-026-36087-z

**Published:** 2026-01-15

**Authors:** Alba Galafat, M. I. Sáez, A. J. Vizcaíno, A. Barany, E. Perera, D. Sánchez-Ruiz, T. F. Martínez, J. Fuentes, J. A. Martos-Sitcha, F. J. Alarcón-López

**Affiliations:** 1 Departamento de Biología y Geología, Escuela Superior de Ingeniería, CEI·MAR-Universidad de Almería, La Cañada de San Urbano, Almería, 04120 Spain; 2 Departamento de Biología, Facultad de Ciencias del Mar y Ambientales, Campus de Excelencia Internacional del Mar (CEI-MAR), Instituto Universitario de Investigación Marina (INMAR), University of Cádiz, Puerto Real, Cádiz, 11519 Spain; 3 Instituto de Ciencias Marinas de Andalucía, Consejo Superior de Investigaciones Científicas (ICMAN-CSIC), Puerto Real, Cádiz, 11519 Spain; 4 LifeBioencapsulation S.L, Parque Científico PITA, El Alquián, Almería, 04131 Spain; 5 Present Address: Department of Genetics, Physiology and Microbiology, Faculty of Biological Sciences, Complutense University of Madrid, Madrid, 28040 Spain

**Keywords:** Digestive enzymes, Electrophysiology, Functional ingredient, Gene expression, Intestinal mucosa, Microalgae, Animal physiology, Ichthyology

## Abstract

Development of more sustainable aquaculture requires alternatives to traditional fishmeal and fish oil in aquafeeds. Among the options, microalgae have emerged as promising functional ingredient, with the potential to provide additional benefits in aquaculture animals. The objective of this piece of research was to assess the effect of the microalgal-based functional ingredients, LB-GUThealth and LB-GREENboost on the intestinal function in juvenile gilthead seabream. Digestive enzyme activities, intestinal mucosa structure and ultrastructure, expression of key intestinal genes, and parameters like transepithelial resistance and permeability were analyzed after administration of feeds supplemented with those algal-based ingredients at two dietary levels (0.5 and 1%) during 91 days. Results indicated improvements in feed utilization efficiency, reflected by an expansion of the absorptive surface of the intestinal mucosa, enlargement of the apical surface of enterocytes and extension of *microvilli* length, together with elevated activity levels of digestive enzymes involved in macronutrient digestion. Additionally, no alterations were observed in basal gene expression related to permeability or the immune system, nor in the bioelectrical parameters associated with the integrity of the intestinal barrier. Results obtained evidenced that the algal-based ingredients tested seem to be useful for improving the intestinal functionality in juvenile gilthead seabream.

## Introduction

The development of sustainable systems capable of ensuring stable fish production and providing healthy and nutritious food to a constantly growing global population is one of the key goals pursued in current aquaculture^1,2^. Since feed is a crucial element for sustainability of modern aquaculture, the success of this sector largely depends on the supply of sufficient quantities of nutritionally balanced aquafeeds^3^. Most of the commercially important aquaculture species require a protein-rich diet, which has traditionally been based on fishmeal and fish oil derived from capture fisheries^4^. However, the decline in fishing production over the past 25 years resulted in higher prices and reduced availability of raw materials for feed^5,6^. Consequently, several scientific studies have been looking into reducing fishmeal and fish oil dependence of the aquaculture industry by replacing them with less expensive and potentially sustainable ingredients, like plant-based feedstuffs^7–9^, or even nutraceuticals or other natural ingredients that enhance the growth performance under conventional formulations^10,11^.

In the same line over the last decade, microalgae have since come up in force as a plausible alternative, claiming their status as a sustainable protein source with ability in aquafeed formulation to replace fishmeal plus other terrestrial plant proteins^12,13^. Practically, the high production cost can always limit industrial implementation; thus, the application of alternative protein is highly compromised by its price. Additionally, the technology used for their large-scale production has not yet been optimised^14^. Despite this drawback, microalgae have been widely studied, not only for their attractiveness as a protein source but also for their potential as a functional ingredient owing to the presence of bioactive compounds exerting beneficial effects on fish health^7,15,16^. Currently, aquafeed design is aimed at covering nutritional requirements for optimal fish development, but also at reducing the environmental impacts derived from intensive aquaculture, and at improving the health status of animals^16–18^. Special attention has been paid for improving intestinal status in fish, given that a healthy intestine determines adequate digestive functionality and correct nutrient absorption and assimilation^19–22^. There is evidence in the literature suggesting that the functional effect would not require high dietary level of microalgae^15^. Thus, making economically viable the use of microalgae at industrial scale, mainly as functional ingredients at low dietary inclusion level. This consideration could further incentivize manufacturing companies’ interest in using microalgae in commercial aquafeeds.

In this sense, incorporating microalgae at dietary low level has demonstrated considerable promise in enhancing growth rates and feed utilisation efficiency^10,23,24^. It has also been shown to improve muscle composition^25–27^, and optimise digestive functionality^21,22,28,29^, and even boost overall fish health, stress resilience, and final product quality. These benefits are of significant interest to both producers and consumers alike^10,22,30,31^. Two microalgal-based functional ingredients, LB-GUThealth (LB_Gh_) and LB-GREENboost (LB_Gb_) from *LifeBioencapsulation* S.L. (a University of Almería’s spin-off (Spain)), have recently demonstrated their ability to enhance feed efficiency and modulate the intermediary metabolism in juvenile gilthead seabream^10^. However, there is currently no available data on their impact on intestinal functionality in this species, leaving open the possibility that any observed phenotypic effects may be linked to changes in gut function. In this sense, the aim of this piece of research is to evaluate the advantages of incorporating two microalgae-based functional ingredients (LB_Gb_ and LB_Gh_) at low or very low dietary levels on the intestinal function in juvenile gilthead seabream. The evaluation focused on various aspects, including digestive enzyme activities, the ultrastructural characteristics of the intestinal mucosa, the changes in the expression of crucial intestinal genes, and parameters such as transepithelial resistance and apparent permeability.

## Materials and methods

### Experimental diets

Four isonitrogenous (46% dry matter) and isolipidic (18% dry matter) aquafeeds were prepared at the CEI·Mar-University of Almeria facilities. The different experimental feeds were named GB5 and GB10, containing 0.5 and 1% of LB-GREENboost ingredient (LB_Gb_), and GH5 and GH10, containing 0.5 and 1% of LB-GUThealth (LB_Gh_). In addition, a control feed (CT) was prepared, with a mixture of standard ingredients that neither include microalgae nor nutraceuticals compounds. LB_Gb_ (containing 57.0% protein, 6.4% fat, 0.4% fibre, 9.4% ash and 7.0% moisture) and LB_Gh_ (containing 56.0% protein, 2.0% fat, 0.2% fibre, 12.7% ash and 8.8% moisture) are concentrated formulations that consist of 80 g and 20 g 100 g^− 1^, respectively, of a hydrolysed blend of *Arthrospira* sp. and *Microchloropsis* sp. The remaining components include choline, calcium carbonate and betaine as excipients. The feeding trials were conducted blindly, with aquafeed’s containers labelled with different colours without any explicit reference to their composition or to which group it represented. This method ensured the elimination of any potential subjectivity or bias during animal feeding. In summary, to prepare the final product, all components were combined in an industrial mixer and processed in a UPZ 100 hammer mill (Hosokawa-Alpine, Augsburg, Germany) to achieve 0.25 mm particle size. Afterward, feeds were processed by extrusion using an Evolum 25 twin-screw extruder (Clextral, France) to produce pellets of 2–3 mm. The configuration of the extruder was setup with a temperature profile of 100 °C, 96 °C, 95 °C, 95 °C, and 90 °C, respectively. The extruded pellets were subjected to drying at 29 °C in a 12 m^3^ chamber (Airfrio, Almeria), after which they were cooled to room temperature, and them coating with oil was performed by using a Pegasus PG-10VC vacuum coater (Dinnissen, The Netherlands). Finally, aquafeeds were placed in airtight bags at − 20 °C until required. The ingredients and proximate composition of the aquafeeds is detailed in Table 1.

**Table 1:** Ingredients and proximal composition (% dry matter) of the experimental aquafeeds.

	CT	GB5	GB10	GH5	GH10
Ingredients					
Fishmeal^1^	20.0	20.0	20.0	20.0	20.0
LB-GREENboost	–	0.5	1.0	–	–
LB-GUThealth	–	–	–	0.5	1.0
Krill meal^2^	2.5	2.5	2.5	2.5	2.5
Soybean protein concentrate^3^	34.2	34.2	34.2	34.2	34.2
Wheat meal^4^	9.4	8.9	8.4	8.9	8.4
Wheat gluten^5^	13.0	13.0	13.0	13.0	13.0
Fish oil^6^	9.2	9.2	9.2	9.2	9.2
Soybean oil^7^	4.4	4.4	4.4	4.4	4.4
Soybean lecithin^8^	1.0	1.0	1.0	1.0	1.0
Betaine^9^	0.5	0.5	0.5	0.5	0.5
Lysine^10^	1.2	1.2	1.2	1.2	1.2
Methionine^11^	0.5	0.5	0.5	0.5	0.5
Vitamins and Minerals^12^	2.1	2.1	2.1	2.1	2.1
Guar gum^13^	1.0	1.0	1.0	1.0	1.0
Alginate^14^	1.0	1.0	1.0	1.0	1.0
Proximal composition (% DM)					
Crude protein	46.2	45.9	45.9	46.0	46.4
Crude lipid	18.4	18.2	18.1	17.9	17.8
Ash	9.9	10.3	10.5	9.9	10.3

**Dietary codes: CT**: control; **GB5**: 0.5% inclusion of LB-GREENboost; **GB10**: 1% inclusion of LB-GREENboost; **GH5**: 0.5% inclusion of LB-GUThealth; **GH10**: 1% inclusion of LB-GUThealth.

^1^12.3% CL, 69.4% CP (Norsildemel, Bergen, Norway).

^2^9% CL, 45% CP (Bacarel, UK).

^3^8% CL, 65% CP (DSM, France).

^4^Local provider (Almería, Spain).

^5^78% CP, 1% ash, 10% moisture (Lorca Nutricion Animal SA. Murcia, Spain).

^6^AF117DHA (Afamsa, Spain).

^7,9,10,11^Lorca Nutricion Animal SA. Murcia, Spain.

^8^P700IP (Lecico, DE).

^12^Lifebioencapsulation SL. Minerals (g kg^− 1^): calcium carbonate, 186 g; iron sulfate, 0.6 g; zinc sulphate 0.75 g; cobalt carbonate, 0.065 g; NaCl, 40 g; cupric sulfate, 0.9 g; KCl, 24.1 g; potassium iodide, 0.05 g; sodium selenite, 0.001 g; manganese oxide, 0.96 g. Vitamins (g kg^− 1^): vitamin D3 (DL-cholecalciferol, 200,000 UI; vitamin A (retinyl acetate), 2,000,000 UI; vitamin K3, 2.5 g; vitamin B1, 3 g; vitamin B2, 3 g; vitamin B9, 1.5 g; vitamin E, 10 g; vitamin B6, 2 g; calcium pantothenate, 10 g; nicotinic acid, 20 g; vitamin B12, 0.001 g vitamin H, 0.3 g; inositol, 50 g; betaine, 50 g.

^13,14^EPSA, Spain.

### Feeding assay and sampling

The feeding assay was conducted at the CTAQUA research centre (El Puerto de Santa María, Cádiz, Spain; Operational Code REGA ES110270000411). Gilthead seabream (*S. aurata*) juveniles were provided by a commercial source (PREDOMAR, Carboneras, Almería). Following a 10-day adaptation phase, 225 gilthead seabream juveniles (12.6 ± 0.1 g average body mass) were randomly allocated in 15 cylindrical 100 L-tanks each, connected to a RAS system, that included biological and physical filters, along with programmable devices for oxygen and temperature control. Oxygen concentration in the outlet water remained above 85% saturation, and the natural photoperiod variations corresponding to the location’s latitude (36° 35′ 06″ N; 06° 13′ 48″ W). Water temperature was maintained at a constant 22 ± 0.5 °C. Experimental diets, as previously described, were provided *ad libitum* to triplicate tanks three times daily, six days a week, from February to May. Fish were weighed and counted every 21 days, and feed intake was registered for assessing the growth performance. No mortality was found in any experimental tanks. Fish were maintained and handled following the guidelines for experimental procedures in animal research of the Ethical and Animal Welfare Committee of the University of Cadiz, according to Spanish (RD53/2013) and European Union (2010/63/UE) legislation. The Ethical Committee from the Autonomous Andalusian Government approved the experiments (Junta de Andalucía reference number 04/04/2019/056). Additionally, this study adhered to the ARRIVE guidelines (https://arriveguidelines.org). We confirm that all experimental procedures were carried out in compliance with these regulations and ethical standards.

At the end of the trial, 24 h-starved juvenile gilthead seabream were sampled. Fish were carefully removed from each tank and placed into a tank containing 200 ppm of clove oil to euthanasia. Entire intestines of 12 fish per treatment (4 per each triplicate tank) were extracted for digestive enzyme analysis. Additionally, intestinal samples were collected from three specimens per tank for visualization using scanning and transmission electron microscopy. For electrophysiological study, 4 specimens from each replicate tank (12 animals per treatment) were sampled for measuring the length from the rectum to the pyloric caeca, and intestinal samples were taken for mounting as outlined below. The remaining animals of were also weighed and measured to assess the overall growth performance of the entire group of animals tested.

### Zootechnical parameters

The feed conversion ratio (FCR) and the intestine length index (ILI) were evaluated as follows: (i) FCR = total feed intake/weight gain; (ii) ILI = (100 × Li)/Lb, being Lb and Li fork body and intestine length, respectively.

### Digestive enzyme extracts

Intestinal tissues from each dietary group were homogenized for obtaining crude extracts for enzyme activities measurement. Each sample was homogenized in distilled water at 4 °C (0.5 g tissue mL^− 1^). Afterward, homogenates centrifuged at 13,000 g for 12 min at 4 °C, and the resulting supernatants were carefully collected and stored at − 20 °C until determination of enzymatic activities.

### Determination of enzyme activities

Determination of trypsin and chymotrypsin activities was performed following methodology of Erlanger et al.^32^. and Del Mar et al.^33^, using 0.5 mM Na-benzoyl-DL-arginine-4-pnitroanilide (BAPNA) and 0.2 mM N-succinyl-(Ala)_2_-Pro-Phe-pnitroanilide (SAPNA) buffered in 50 mM Tris-HCl with 10 mM CaCl_2_ as substrates, respectively. For both enzymes, one activity unit (U) was defined as the enzyme amount required to release 1 µmol of p-nitroanilide (pNA) min^− 1^, measured spectrophotometrically at 405 nm, using 8800 M cm^− 1^ as extinction coefficient. For its part, activity of total alkaline protease was measured following Alarcón et al.^34^. One unit of activity was stablished as the enzyme amount that liberated 1 µg of tyrosine min^− 1^, using 0.008 µg^− 1^ mL^− 1^ cm^− 1^ as extinction coefficient for tyrosine at 280 nm. Finally, enzymatic activities related to the intestine brush border, specifically leucine aminopeptidase and alkaline phosphatase, were assessed following the protocols of Pfleiderer^35^ and Bergmeyer^36^, respectively. The enzyme amount that releases 1 µmol p-nitroanilide min^− 1^, considering 8800 M cm^− 1^ as extinction coefficient, measured spectrophotometrically at 405 nm was defined as unit of enzymatic activity. For alkaline phosphatase activity, one unit released 1 µg nitrophenyl min^− 1^, with an extinction coefficient of 17,800 M cm^− 1^ at 405 nm.

### Ultrastructural study of the intestine mucosa

**Fig. 1:**
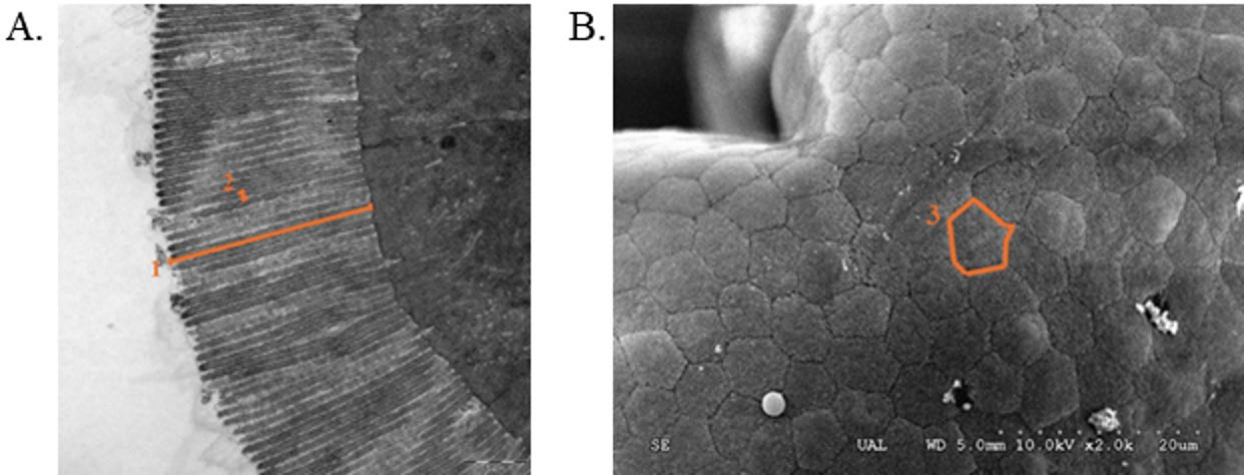
Details of the measurements performed on intestinal *microvilli* and enterocyte apical area, used to estimate the total absorption surface. (**A**) 1. *Microvilli* length (ML); 2. *Microvilli* diameter (MD); (**B**) 3. Enterocyte apical area (EA).

First, intestine sections for TEM analysis were fixed in a mixture of 40 g L^− 1^ of formaldehyde 25 g L^− 1^ of glutaraldehyde and in phosphate buffer saline (PBS) at pH 7.5 (4 h, 4 °C). After fixation, samples were rinsed with PBS for 20 min and fixed in 20 g L^− 1^ of osmium tetroxide solution. Sequently, intestine sections were dehydrated by sequential immersion in ethanol solutions, gradually increasing from 50% to 100% (v/v). After dehydration, samples were infiltrated with a solution of Epon resin and 100% ethanol (1:1) for 2 h, followed by embedding in pure Epon resin and polymerisation at 60 °C. The sections were mounted onto 700 Å copper grids and stained using lead citrate and uranyl acetate Finally, grids were examined using a Zeiss 10 C TEM at 100 Kv (Carl Zeiss, Spain). For SEM samples preparation, intestines were washed 1% S-carboxymethyl-L-cysteine. Then, samples were fixed in 4% PBS-buffered formaldehyde at pH 7.2 for 24 h. After fixation, intestine sections were rinsed, dehydrated in ethanol and then dried using a CDP 030 critical point dryer (Leica Microsystems, Spain). Then, samples were mounted with PELCO colloidal graphite (Ted Pella INC, USA), gold-coated using a SCD 005 Sputter Coater (Leica Microsystems) and observed using a HITACHI S-3500 scanning electron microscope (Hitachi High-Technologies Corporation, Japan). TEM and SEM images were measured using the UTHSCSA ImageTool software (University of Texas Health Sciences Centre, USA). TEM images were used for measuring length of *microvilli* (ML), diameter of *microvilli* (MD), and the number of *microvilli* per micrometre, and SEM images for calculating the apical area (EA) of enterocytes (Fig. 1). Finally, total enterocyte absorption surface (TAS) was calculated using the data from both types of imaging, as described previously by Vizcaíno et al.^37^.

### Voltage clamp in Ussing chambers

The sections of anterior intestine of each fish were carefully excised and ubicated on a tissue holder with an area of 0.25 cm², as described by Estensoro et al.^38^. They were then positioned between two half-chambers, each containing 2 mL of a saline solution (160 mM L^− 1^ NaCl, 2 mM L^− 1^ NaH_2_PO_4_, 5 mM L^− 1^ NaHCO_3_, 1 mM L^− 1^ MgSO_4_, 1.5 mM L^− 1^ CaCl_2_, 3 mM L^− 1^ KCl, 5.5 mM L^− 1^ glucose and 5 mM L^− 1^ HEPES, pH 7.8). Throughout the experiment, intestinal tissue was supplied with a gas mixture of 99.7% O_2_ + 0.3% CO_2_ at 22 °C. Short-circuit current (Isc, µA cm^− 2^) was assessed by clamping the epithelia to 0 mV. Current injections and voltage clamping were managed using VCC MC6 and VCC MC8 amplifiers (Physiologic Instruments, San Diego, USA). Data were continuously recorded using the Labscribe3 software, along with an IWorx188 data acquisition system, for a period of 90 min from the mounting time. Epithelial resistance (Rt, Ω cm²) was determined manually by applying a +/-1 mV bilateral pulse for 3 s per min and calculating the resulting current deflections using Ohm’s law. The apical side was designated as the ground, with negative currents indicating absorption and positive currents indicating secretion.

### Permeability assay

After allowing 20 min for tissue stabilization, the buffered saline solution was changed with fresh, well-gassed solution, ensuring a volume of 2 mL per chamber. A concentrated stock solution of FITC-dextran (average molecular weight 4,000, Sigma, Madrid) at 100 mg mL^− 1^ was prepared and added to the apical chamber to reach 0.5 mg mL^− 1^. A 0.2 mL sample was then taken from either the basolateral or apical compartment after 5 min of mixing for establishing the baseline (time zero). After one-hour, additional samples were collected from both compartments and stored in fresh vials. Fluorescence readings were taken using a BioTek SynergyTM 4 Multi-Mode Microplate Reader (BioTek^®^ Instruments, Winooski, USA), using 492 nm for excitation and 520 nm for emission. The apparent permeability (Papp) was calculated using the formula: Papp = (V * dC) / (A * C0 * dT), being V the volume in the receiving chamber, A the tissue surface area, C0 the initial concentration in the donor compartment, and dC/dT is the rate of change in the concentration of FITC (ng s^− 1^) in the receiving chamber. Concentration standards ranging from 0.2 to 2000 ng mL^− 1^ were used to determine concentration in both compartments.

### mRNA extraction and qPCR analysis

Anterior intestines, preserved in RNAlater^®^ (Invitrogen Life Technologies; Waltham, MA, USA), were processed individually using a glass homogeniser. RNA was extracted using the Total RNA Kit I (E.Z.N.A, Omega Bio-tek, USA), incorporating a DNase treatment with the DNA-free Kit (RNase-Free DNase I Set, Omega Bio-tek) as per the manufacturer’s instructions. RNA purity and concentration were evaluated using a Nanodrop One (Thermo Scientific, Waltham, USA), with only samples with an A260/A280 ratio ranged from 1.9 to 2.2 were selected for cDNA synthesis. Reverse transcription was performed with the RevertAid First Strand cDNA Synthesis Kit (Thermo Scientific #K1691, UK) using 500 ng of RNA in a 20 µL. Real-time qPCR amplifications were conducted in duplicates, in 6 µL, containing 3 µL of SsoFast EvaGreen Supermix (Bio-Rad, UK) and 0.25–6.25 ng of total cDNA. Prior to the experiment, optimal primer concentrations (150 nM for 18 S; 250 nM for others) and annealing temperatures (58 °C for 18 S; 60 °C for others) were determined. Real-time amplifications were conducted using a BIO-RAD CFX Connect system (Bio-Rad Laboratories, Hercules, USA) on 384-well plates, following the outlined procedure: an initial denaturation step at 95 °C for 35 s, followed by 40 cycles of 15 s at 95 °C for denaturation, 10 s at 58–60 °C for annealing and extension, and a final melting curve from 60 °C to 95 °C, increasing by 0.5 °C every 5 s. The melting curve confirmed the presence of a single PCR product and ruled out primer-dimer formation. To determine primer efficiency, a standard curve was constructed using tenfold serial dilutions (ranging from 10 ng to 0.001 pg) of a cDNA pool from all samples. For each primer pair, linearity (R^2^ > 0.98) and amplification efficiency (90–110%) were confirmed. Gene expression was quantified by using the ΔΔCT method (Livak and Schmittgen, 2001) and normalised to one reference gene (eef1a) as described by Vandesompele^39^. Control reactions, consisting of RNase-free water (NTC) and RNA (NRT), were included to confirm the absence of genomic DNA contamination and primer dimers. Furthermore, the pGEM-T Easy vector (Promega, Madison, USA) was used for cloning all PCR products, and their identity was verified through Sanger sequencing at CCMar (Faro, Portugal). The sequences of primers employed for qPCR are detailed in Table 2.

**Table 2:** Specific primers utilised for real-time qPCR expression analysis.

Gene name	Symbol	Acc. no.		Primer sequences (5’-3’)
Claudin3	*cldn3*	KF861991.1	F	TGAGGGTGAACTGAGGAACA
			R	TGGAAGACAAAGAGCCTACG
Claudin4	*cldn4*	XP_030292963	F	CCACACCATCATCCGAGAC
			R	CTCATCTTTAGGAGGGCAGTTG
Claudin4-like	*cldn4l*	XP_030264161	F	CTCTGGCTCTGGGTGTCCTC
			R	CTGATGATGGAGTGGGCAGT
Claudin5	*cldn5*	UIP35178	F	CTGCTGTGCTGCTCCTGTC
			R	GTTCTGCGTGGCTCTCTTG
Claudin7b	*cldn7b*	UIP35180	F	TACGCTCACGACATCATCCA
			R	CCAACTACAGCCAGGAAAGC
Claudin15	*cldn15*	KF861993.1	F	AAACCCACTTTGTGATTGCA
			R	TGTTTGACCTTCCCCTTACAA
Claudin24	*cldn24*	UIP35181	F	CATTGTTCTGGCGTGTATCG
			R	TTGTTGGTCCCTTGACTTGG
Occludin-like	*oclnl*	XP_030259382	F	TGTTGTTGTTTCGGAGAGAGC
			R	CGACGACTGTTCTTGTCAGC
Occludinb	*oclnb*	JQ692876.1	F	TCGCGTTGTTGATGCTAATA
			R	TGGTTGACGAACACTCCTGA
Occludinc	*oclc*	KF861990.1	F	AGAAACAGGCAATGAACTCG
			R	GGTCGGCGTCAAACTCTCT
Immunoglobulin M	*igm*	JQ811851	F	ACCTCAGCGTCCTTCAGTGTTTATGATGCC
			R	CAGCGTCGTCGTCAACAAGCCAAGC
CD4-full	*cd4*	AM489485	F	TCCTCCTCCTCGTCCTCGTT
			R	GGTGTCTCATCTTCCGCTGTCT
Interleukin-10	*il10*	JX976621	F	AACATCCTGGGCTTCTATCTG
			R	GTGTCCTCCGTCTCATCTG
Toll-like receptor 9	*tlr9*	AY751797	F	GCCTTCCTTGTCTGCTCTTTCT
			R	GCCGTAGAGGTGCTTCAGTAG
Elongation factor 1-alpha^1^	*eef1a*	AF184170	F	AGAGGCTGTCCCTGGTGA
			R	TGATGACCTGAGCGTTGAAG

GenBank accession number (Acc. No.) referred to the unique sequence identifier in the gene assembly.

^1^Reference gene.

### Statistical analysis

All experiments were conducted with a minimum of three repetitions and three replicates per condition. Outliers were identified using the ROUT method (Q = 1%) and removed from subsequent analyses. Data are presented as mean ± SEM. To compare the means, a two-way ANOVA was performed with a significance level set-up at 5% (*P <* 0.05), followed by a multiple comparison test. Percentage data were arcsine (×^1/2^)-transformed and logit(y) (for genes *cldn3*,* cldn4*,* cldn4l*,* cldn5*,* cldn15*,* cldn24*,* oclnl*,* oclnb*,* oclnc*,* igm*,* cd4*,* il10 and tlr9*), and normality (Shapiro-Wilk test) and homoscedasticity (Levene test) were assessed. If the data violated the assumptions of ANOVA, a Kruskal-Wallis one-way analysis of variance on ranks was performed. Additionally, the influence of categorical variables, namely “*FI*” (functional ingredient) and “*dose*,” was evaluated for each numerical parameter through a generalized linear model (GLM analysis) to identify relationships between measured variables and predictive factors. All statistical analyses were conducted using Statgraphics Plus 18 software (USA).

## Results

### Growth performance and intestine length index

**Fig. 2:**
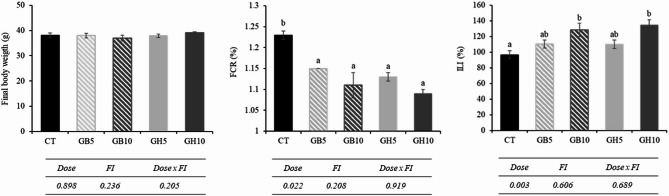
Growth performance of juvenile gilthead seabream fed the different experimental diets. CT: control; GB5: 0.5% inclusion of LB-GREENboost; GB10: 1% inclusion of LB-GREENboost; GH5: 0.5% inclusion of LB-GUThealth; GH10: 1% inclusion of LB-GUThealth. Values are mean ± SEM.

Growth performance and intestinal length index of *S. aurata* juveniles fed experimental diets are shown in Fig. 2. No mortality was detected throughout the duration of the feeding trial. In addition, all fish groups exhibited continuous growth, increasing from 12 to 13 g to 37–39 g, with a significant reduction in feed conversion ratio (FCR; *P <* 0.001, one-way ANOVA), which decreased from 1.23 in the control group (CT) to 1.13–1.09 in fish fed both functional ingredients (LB_Gb_ and LB_Gh_) at inclusion levels of 0.5% and 1%. Intestinal length index (ILI) showed a dose-dependent increase in fish fed both functional ingredients (*P =* 0.003, GLM).

### Digestive enzyme activity

**Fig. 3:**
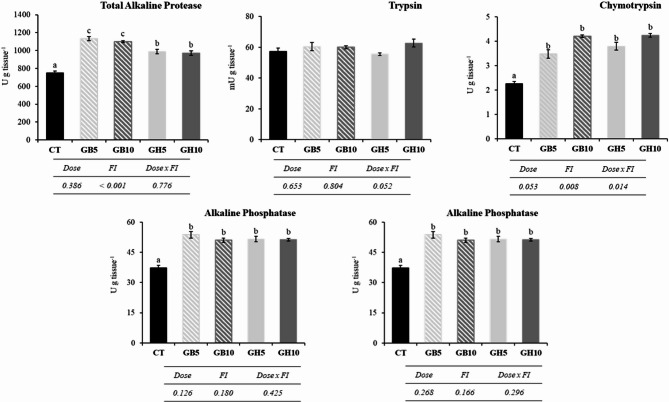
Activity levels of digestive enzymes in gilthead seabream (*S. aurata*) juveniles fed with different experimental diets. Dietary codes: CT: control; GB5: 0.5% inclusion of LB-GREENboost; GB10: 1% inclusion of LB-GREENboost; GH5: 0.5% inclusion of LB-GUThealth; GH10: 1% inclusion of LB-GUThealth. Values expressed as mean ± SEM (*n* = 12). Lowercase letters indicate significant differences among dietary treatments.

The activity of intestinal enzymes is shown in Fig. 3. Overall, with exception of trypsin, level of activity in all enzymes increased in specimens fed diets supplemented with functional ingredients. More specifically, in total alkaline protease activity, the highest values were obtained in specimens fed diets containing LB_Gb_ ingredient, regardless dose used, followed by fish fed GH5 and GH10 dietary treatments (*P <* 0.001, one-way ANOVA). Chymotrypsin activity was significantly lower in CT group compared to the rest of dietary groups (*P <* 0.001, one-way ANOVA). Furthermore, a dependence on the type of ingredient administered (*P* = 0.008, GLM) was observed, as well as the interaction between the type of FI and the dose used (*P* = 0.014, GLM). Leucine aminopeptidase and alkaline phosphatase activities significantly increased in fish fed with diets supplemented with functional ingredients (*P <* 0.001 in both cases, one-way ANOVA). However, the effect was not influenced by type of FI and dose used.

### Ultrastructural study of the intestinal mucosa

**Fig. 4:**
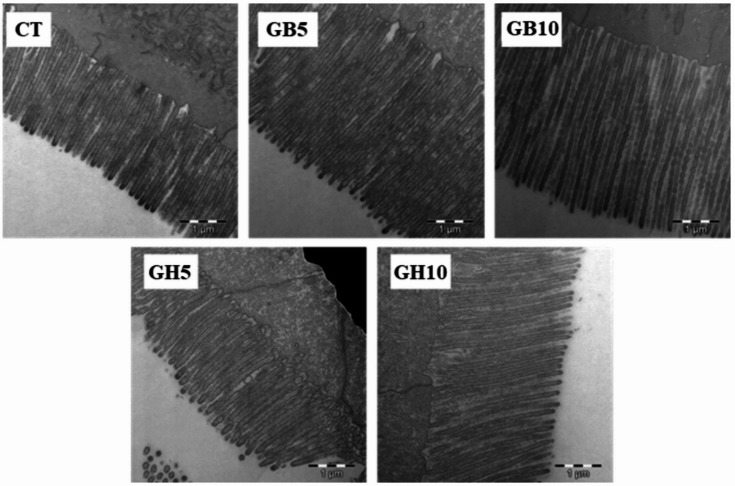
TEM details of the apical surface of the enterocytes in gilthead seabream juveniles fed the different experimental diets (TEM. Scale: 1 μm). **Dietary codes: CT**: control; **GB5**: 0.5% inclusion of LB-GREENboost; **GB10**: 1% inclusion of LB-GREENboost; **GH5**: 0.5% inclusion of LB-GUThealth; **GH10**: 1% inclusion of LB-GUThealth.

**Fig. 5:**
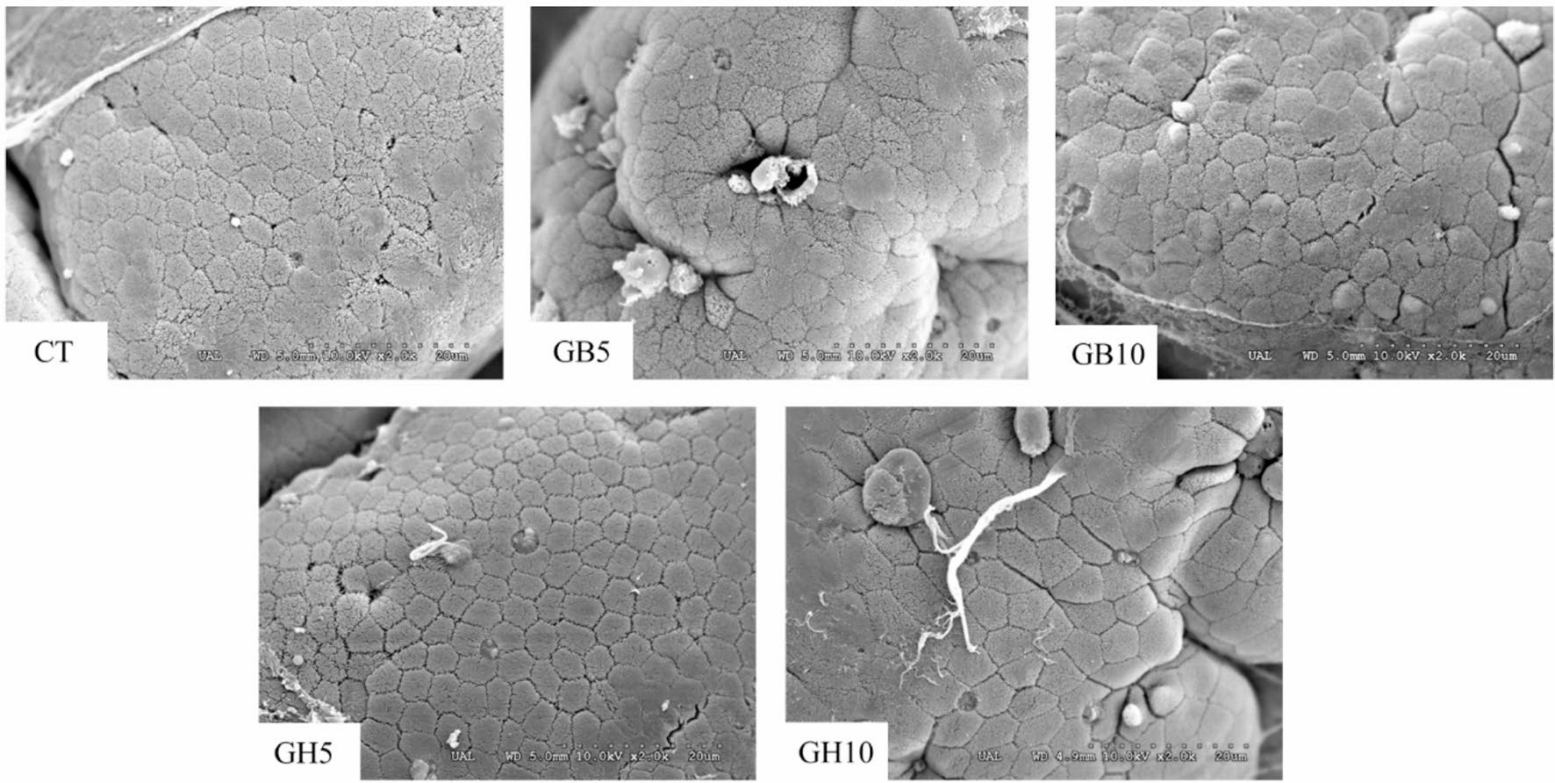
SEM details of the enterocyte apical surface in gilthead seabream juveniles fed the different experimental diets (SEM. Scale: 20 μm). Dietary codes: CT: control; GB5: 0.5% inclusion of LB-GREENboost; GB10: 1% inclusion of LB-GREENboost; GH5: 0.5% inclusion of LB-GUThealth; GH10: 1% inclusion of LB-GUThealth.

Examination of TEM and SEM electron microscopy images revealed a normal intestinal mucosa with regularly arranged *microvilli* on the surface of enterocytes, and no signs of structural damage (Figs. 4 and 5).

Ultrastructural analysis (Table 3) showed that dietary inclusion of the functional ingredients affected the intestinal mucosa morphology, particularly in fish fed with the higher dose. Specifically, a significant increase (*P <* 0.001, one-way ANOVA) was observed in *microvilli* length and apical area of the enterocytes in specimens fed with GB5, GH5 and GH10, compared to CT group. *Microvilli* diameter was significantly smaller in fish fed LB-GREENboost-supplemented diets, while fish fed diets supplemented with LB-GUThealth showed larger diameter compared to CT group. Finally, total absorption surface per enterocyte significantly increased in animals of GB5, GB10 and GH10 treatments, compared to CT, while GH5 group did not show difference. Furthermore, a clear influence of the ingredient dose on *microvilli* length and total absorption surface was found. Similarly, the type of ingredient supplied significantly influenced both the length and diameter of the *microvilli*, as well as the apical area of the enterocytes and total absorption surface.

**Table 3:** Morphometric analysis of intestinal mucosa of Gilthead seabream juvenile fed with the different experimental diets.

	CT	GB5	GB10	GH5	GH10	*P*	Dose	FI	Dose x FI
MD (µm)	0.11 ± 0.01^a^	0.11 ± 0.01^a^	0.11 ± 0.02^a^	0.13 ± 0.01^c^	0.12 ± 0.01^b^	< 0.001	0.0555	< 0.001	0.016
ML (µm)	2.62 ± 0.05^b^	3.04 ± 0.07^d^	3.53 ± 0.04^e^	1.93 ± 0.03^a^	2.83 ± 0.05^c^	< 0.001	< 0.001	< 0.001	0.021
EA (µm^2^)	12.87 ± 0.18^a^	14.97 ± 0.20^b^	17.56 ± 0.25^c^	13.08 ± 0.20^a^	21.31 ± 0.27^d^	< 0.001	0.646	< 0.001	< 0.001
TAS (µm^2^)	617.45 ± 8.77^a^	1003.19 ± 13.03^b^	1254.26 ± 24.03^c^	623.53 ± 10.07^a^	1182.54 ± 15.10^c^	< 0.001	< 0.001	< 0.001	< 0.001

Values are mean ± SEM (*n* = 50). MD: *microvilli* diameter; ML: *microvilli* length; EA: enterocyte apical area; TAS: total enterocyte absorption surface. **Dietary codes: CT**: control; **GB5**: 0.5% inclusion of LB-GREENboost; **GB10**: 1% inclusion of LB-GREENboost; **GH5**: 0.5% inclusion of LB-GUThealth; **GH10**: 1% inclusion of LB-GUThealth. Values in the same row with different lowercase letters indicate significant differences between treatments (*P* < 0.05). Factors evaluated in the statistical analysis: diet, dose and FI (functional ingredient).

### Voltage clamp in Ussing chambers and permeability assay

**Fig. 6:**
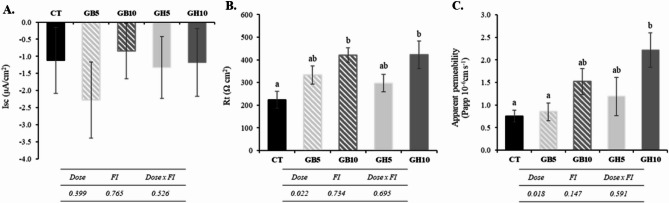
Intestinal physiology parameters in relation to (**A**) short-circuit current (Isc), (**B**) epithelial resistance (Rt), and (**C**) apparent permeability in gilthead seabream juveniles fed with the different experimental diets. Data are the mean ± SEM (*n* = 12). Lowercase letters indicate significant differences between treatments. Dietary codes: CT: control; GB5: 0.5% inclusion of LB-GREENboost; GB10: 1% inclusion of LB-GREENboost; GH5: 0.5% inclusion of LB-GUThealth; GH10: 1% inclusion of LB-GUThealth.

At the electrophysiological level, the short-circuit current (Isc) did not reveal significant differences among treatments, always remaining with negative values (Isc < 0; Fig. 6A). The negative Isc values found in all fish indicate a secretory function, reflecting the prevailing electrolyte transport across the epithelium. On the other hand, the epithelial resistance (Rt, Fig. 6B) showed a significant and dose-dependent increase, reaching a significant increase of ~ 200% in fish fed with the highest levels of supplementation (GB10 and GH10) compared to the control group (CT). Apparent permeability (Papp, Fig. 6C) showed an equally dose-dependent increase in fish fed with both compounds, but statistically significant differences were only found in those belonging to the GH10 group.

### mRNA expression

The results of gene expression profiling for intestine-selected genes are summarized in Table 4. Dietary supplementation did not significantly affect the mRNA expression levels of any assessed genes. However, the ingredient had a significant effect on *cldn4l*, while the dose significantly influenced *oclnb*, both of which are permeability-related genes. Additionally, trends were observed for the effect of the ingredient on *cldn4l*, *cldn5*, and *igm*. No significant differences were detected among treatments in the post hoc multiple comparisons.

**Table 4:** Relative mRNA expression of different claudins (*cldn*), occludins (*ocld*) and immune-related (*igm*, *cd4*, *il10*, tlr9) genes in Gilthead seabream juveniles fed with the different experimental diets.

	CT	GB5	GB10	GH5	GH10	*P*	Dose	FI	Dose x FI	Trans.
*cldn3*	1.00 ± 0.35	1.10 ± 0.32	0.85 ± 0.22	0.66 ± 0.19	0.96 ± 0.20	*0.234*	*0.409*	*0.350*	*0.827*	sin(y)
*cldn4*	1.00 ± 0.59	1.09 ± 0.54	0.82 ± 0.24	0.96 ± 0.42	0.83 ± 0.30	*0.667*	*> 0.999*	*0.750*	–	rank(y)
*cldn4l*	1.00 ± 0.10	0.92 ± 0.06	0.78 ± 0.06	0.71 ± 0.04	1.01 ± 0.12	*0.079*	*0.646*	*0.096*	*0.042*	1/y
*cldn5*	1.00 ± 0.13	0.86 ± 0.11	0.81 ± 0.13	0.99 ± 0.07	1.20 ± 0.14	*0.417*	*0.831*	*0.079*	*0.621*	log(y)
*cldn7b*	1.00 ± 0.12	0.80 ± 0.05	0.85 ± 0.06	0.89 ± 0.07	1.04 ± 0.12	*0.301*	*0.990*	*0.653*	*0.994*	arcsin(y)
*cldn15*	1.00 ± 0.06	1.02 ± 0.11	0.95 ± 0.06	0.91 ± 0.04	1.27 ± 0.12	*0.178*	*0.256*	*0.399*	*0.259*	1/y
*cldn24*	1.00 ± 0.14	1.49 ± 0.45	1.70 ± 0.26	1.41 ± 0.36	1.57 ± 0.39	*0.059*	*0.563*	*0.102*	*0.770*	log(y)
*oclnl*	1.00 ± 0.13	0.60 ± 0.08	0.76 ± 0.10	0.64 ± 0.04	0.75 ± 0.11	*0.056*	*0.950*	*0.228*	*0.482*	arcsin(y)
*oclnb*	1.00 ± 0.16	0.74 ± 0.08	0.92 ± 0.19	0.70 ± 0.15	0.66 ± 0.07	*0.153*	*0.864*	*0.051*	*0.830*	sin(y)
*oclnc*	1.00 ± 0.12	1.01 ± 0.16	1.04 ± 0.15	0.91 ± 0.06	0.90 ± 0.11	*0.263*	*0.907*	*0.727*	*0.781*	sin(y)
*igm*	1.00 ± 0.17	0.74 ± 0.15	0.66 ± 0.14	0.83 ± 0.14	0.68 ± 0.10	*0.098*	*0.623*	*0.064*	*0.812*	log(y)
*cd4*	1.00 ± 0.10	1.12 ± 0.20	0.84 ± 0.09	1.08 ± 0.10	1.04 ± 0.13	*0.246*	*0.214*	*0.466*	*0.582*	1/y
*il10*	1.00 ± 0.29	1.18 ± 0.39	0.87 ± 0.18	0.86 ± 0.16	1.06 ± 0.26	*0.347*	*0.899*	*0.860*	*0.897*	log(y)
*tlr9*	1.00 ± 0.09	0.90 ± 0.12	2.00 ± 0.63	1.23 ± 0.22	1.14 ± 0014	*0.379*	*0.987*	*0.809*	*0.649*	logit(y)

**Dietary codes: CT**: control; **GB5**: 0.5% inclusion of LB-GREENboost; **GB10**: 1% inclusion of LB-GREENboost; **GH5**: 0.5% inclusion of LB-GUThealth; **GH10**: 1% inclusion of LB-GUThealth. Data are presented as the mean ± SEM, calculated from untransformed data (*n* = 9–12).

## Discussion

Over the last decades, the development of aquaculture has increasingly focused on improving feeding practices, in which functional ingredients have emerged as valuable tools offering significant benefits for enhancing animal health and performance^40,41^. Numerous studies have highlighted the benefits of functional ingredients in mitigating stress^42,43^, however, their implementation on a large scale still requires further research. Reducing fishmeal in carnivorous fish diets without compromising growth and health remains a major challenge^44–46^. The use of functional ingredients could help overcome digestive problem associated with high inclusion dietary levels of plant proteins^16,47–49^.

Since the intestine is the primary organ affected by dietary changes and plays a central role in nutrient absorption and overall health, prior evaluation of dietary ingredients is essential to optimize inclusion levels and ensure their effectiveness. The use of functional ingredients could help overcome digestive problems associated with high dietary inclusion levels of plant proteins^16,47–49^. In this context, the present study evaluated the effect of two functional ingredients, LB-GREENboost and LB-GUThealth, previously described by Perera et al.^10^, on intestinal function in juvenile gilthead seabream using different experimental approaches. In their study, Perera et al.. demonstrated clear improvements in physiological traits related to intermediary metabolism in juvenile gilthead seabream. Specifically, dietary administration with LB_Gb_ and LB_Gh_ did not impair the growth performance or survival throughout the experimental period, although it resulted in reduced feed intake, potentially implying lower production costs and increasing competitiveness for this species. Such outcomes may be achieved by gut modulation (e.g. elongation of the fish intestine) and by orchestrating different metabolic physiological processes^10^, and by improving the intestinal functionality, as confirmed in the present study.

The health of the fish intestinal tract is associated with effective digestive function, as well as with proper nutrient absorption and assimilation^19,40^. In this context, a favourable feed conversion ratio is strongly linked to the physiological ability of fish to digest and utilize dietary nutrients, which depend on the presence of an adequate set of digestive enzymes^50^. Fish have the capacity to adapt their digestive enzyme profiles to dietary changes^51^. These adaptive mechanisms can occur rapidly and provide valuable insight into the effects of diet on intestinal function and integrity, particularly when feeds are formulated with alternative protein sources^52^.

Pancreatic proteases are essential for a proper digestive process, being primarily responsible for protein hydrolysis into smaller peptides and free amino acids^53^. Their activity levels are commonly used as indicator of the nutritional condition of fish^34^. Overall, the results obtained in the present study evidenced increased total alkaline protease and chymotrypsin activities in fish fed diets supplemented with the functional ingredients. Similar findings were reported by Abdel-Tawwab et al.^28^. and Galafat et al.^20^. , who observed increased digestive enzyme activities following administration of low dietary levels of *Scenedesmus quadricauda* and *Arthrospira* sp. in Nile tilapia (*Oreochromis niloticus*) and gilthead seabream (*S. aurata*), respectively. These studies demonstrated that low dietary level of microalgae can modulate pancreatic secretion in fish. The present study also demonstrated improved digestive function in juvenile gilthead seabream following low dietary inclusion of microalgae. Specifically, the functional ingredients consisted of 20% and 80% microalgae, respectively, corresponding to dietary algal supplementation levels of 0.1% and 0.8%, respectively. However, trypsin activity was not affected, suggesting a differential regulation mechanism for individual digestive enzymes. It can be hypothesised that chymotrypsin and total alkaline protease activities may be modulated by the peptides or bioactive compounds provided by the functional ingredients, whereas trypsin activity may require alternative regulatory stimuli as detailed other studies^51,54,55^.

Intestinal brush border enzymes play a crucial role during the final phase of digestion and nutrient absorption^20,52^. Specifically, leucine aminopeptidase hydrolyses peptides into free amino acids, which are subsequently absorbed by the intestinal mucosa^21^. In the present study, both functional ingredients increased leucine aminopeptidase activity, which may be associated with enhanced nutrient absorption efficiency, as supported by the electrophysiological analysis results. Similar findings were reported by Galafat et al.^20^ and Sáez et al.^22^, who evaluated low dietary inclusion levels of *Arthrospira* sp. (2%) and *Microchloropsis gaditana* (2.5%), respectively, in juvenile gilthead seabream. Alkaline phosphatase is commonly used as a marker of intestinal structural integrity because it is localized in the brush border membrane^52^. This enzyme hydrolyses phosphoester bonds in a wide range of organic compounds, including proteins, lipids, and carbohydrates, thereby releasing phosphate^52^. The results of the present study showed increased alkaline phosphatase activity in fish fed diets supplemented with the functional ingredients. Vizcaíno et al.^37^. and Galafat et al.^21^. reported similar effects when *Scenedesmus almeriensis* and *Arthrospira* sp., respectively, were incorporated into the diets of gilthead seabream juveniles. Overall, the findings of the present study indicate that these functional ingredients can improve the intestinal absorptive capacity in juvenile *S. aurata*.

It is well established that the ultrastructure of the intestinal mucosa is essential both for ensuring adequate nutrient absorption and for providing a physical and immunological barrier against potential pathogens. Accordingly, alkaline phosphatase, an enzyme tightly bound to the hydrophobic core of the intestinal *microvilli* membrane^52^, is commonly used as a marker of both the intestinal functionality and mucosal integrity. Thus, the increased alkaline phosphatase activity observed in the present study may be associated with morphological improvements of the intestine, as reflected by the ultrastructural analysis of the intestinal mucosa and the transepithelial resistance values recorded. Our findings revealed increased *microvilli* length and enterocyte apical area in the GB5, GB10, and GH10 groups, resulting in an increased absorptive surface area, which is consistent with the elevated alkaline phosphatase activity observed. Several studies have also reported increased absorptive surfaces of the intestinal mucosa in fish following dietary microalgae supplementation^29,56–58^. These results support the hypothesis that functional ingredients could represent an effective strategy for optimizing intestinal function in aquaculture, however, further studies are required to evaluate their effects under different conditions and across species.

Furthermore, it is crucial to investigate and discuss the relationship between electrophysiological and gene expression data within the context of intestinal architecture and permeability. Previous findings indicate that the inclusion of plant proteins or algae-derived raw materials in aquafeeds for carnivorous species can influence intestinal barrier function, selectivity, and integrity. This is evidenced by changes in bioelectrical properties assessed through epithelial electrophysiology, as well as microbiota analysis, and molecular markers^59,60^. The negative Isc values observed in all dietary groups could be associated with absorptive function, reflecting proper epithelial functionality and the absence of inflammation or enteritis, which are commonly linked to current inversions. Regarding Rt, this parameter is considered a measure of tissue integrity and represents the electrical expression of barrier function in epithelial systems^61^. The observed increase in Rt indicated appropriate intestinal mucosa integrity across all dietary treatments, as supported by the morphological and enzymatic analysis, and a general improvement in transepithelial electrical resistance, particularly in specimens fed the highest dietary dose of the functional ingredients (GB10 and GH10). Although apparent permeability (Papp) increased significantly in fish fed the GH10 diet, these changes cannot be associated with an impaired intestinal barrier function, given the above-mentioned increase in transepithelial resistance in intestinal samples from this group. In contrast, other studies have reported impaired intestinal barrier function with increased paracellular permeability and decreased transepithelial resistance^62^. Nevertheless, since neither decreased Rt nor detrimental structural effects were observed in the present study, it can be ruled out that changes in permeability may indicate tissue damage. Further research is required to ascertain the effects of the functional ingredients on intestinal permeability using different paracellular tracers.

The observed changes in permeability in the current study could also be attributed to alterations in intestinal tight junctions (TJs) proteins. TJs establish crucial barriers that separate the paracellular space from the intestinal lumen, as well as the apical and basolateral regions of the plasma membrane^63^. These junctions regulate the paracellular transport of water, ions, and small molecules, with occludins and claudins acting as key structural components. These proteins are essential for maintaining TJ functionality and directly influence transepithelial resistance (TER) in the intestinal epithelium. However, changes in transepithelial resistance (TER) and apparent permeability (Papp) may vary depending on tight junction regulation, reflecting their distinct biological roles. While both parameters assess barrier function, they are influenced by different molecular pathways and can respond independently to physiological or experimental stimuli. Alterations in TER are closely associated with changes in TJ protein expression^64^. Accordingly, to investigate the molecular mechanisms underlying diet-induced changes in tissue resistance following nutraceutical supplementation, qPCR analyses targeting claudins and occludins were performed. The expression of these proteins determines whether the epithelial barrier exhibits a “leaky” or “tight” function^65^. Specifically, occludin downregulation has been linked in some studies to decreased TER in Caco-2 monolayers^66^. Moreover, molecular regulation of intestinal functionality and TJ proteins is essential for maintaining intestinal barrier integrity, which can be assessed by epithelial electrophysiology. Under the experimental conditions tested, none of the functional ingredients or dietary doses evaluated elicited significant changes in basal gene expression patterns, intestinal architecture, or immune-related genes.

Taken together, these findings highlight the importance of understanding both the physiological and molecular responses to dietary changes, as these determine nutrient absorption and overall gut health in fish. Notably, after the 3-month feeding trial, intestinal functionality exhibited both phenotypic changes (e.g., intestinal elongation and increased microvilli length) and mechanistic changes (e.g., increased transepithelial resistance and apparent permeability) under the experimental conditions tested. It is also worth emphasizing that the observed effects of nutraceuticals supplementation occurred in diets formulated similarly to premium commercial diets, characterized by high proportion of marine-derived raw materials. Future studies should evaluate these nutraceuticals under more sustainable feeding regimes (e.g., plant-based or alternative protein sources) to determine their ability to improve intestinal health and function in aquaculture species. In addition, retrospective molecular analyses may help clarify the underlying mechanisms involved and determine the optimal timing to enhance gut functionality during dietary interventions.

Overall, the results suggest that the dietary inclusion of LB-GREENboost and LB-GUThealth improves intestinal functionality and nutrient utilization in juvenile gilthead seabream. These functional ingredients may enhance absorptive capacity by increasing the apical surface area of enterocytes and the *microvilli* length, improve epithelial selectivity for nutritionally valuable compounds, and stimulate the activity of key digestive enzymes, without compromising basal intestinal permeability or affecting immune-related gene expression. Collectively, these findings indicate that functional ingredients could contribute to more efficient feed utilization and healthier gut physiology, addressing critical challenges in sustainable aquaculture, such as optimizing plant-based diets for carnivorous species. Future studies should focus on elucidating the molecular mechanisms underlying these effects, determining optimal inclusion levels, and evaluating their impact at different developmental stages and across species. Overall, the results obtained support the hypothesis that microalgae-derived functional ingredients represent a promising strategy for enhancing intestinal health and digestive efficiency in aquaculture species, providing an applicable approach for improving nutritional practices.

## Data Availability

The datasets generated and analyzed during the current study have been submitted to the Gene Expression Omnibus (GEO) repository (record GSE289127) with the primary accession code oroxcacsxtcbloj.

## Abbreviations

EA: Enterocyte apical area
FCR: Feed conversion ratio
ILI: Intestine length index
LB_Gb_: LB-GREENboost functional ingredient
LB_Gh_: LB-GUThealth functional ingredient
MD: Microvilli diameter
ML: Microvilli length
Papp: Apparent permeability
SEM: Scanning electron microscopy
TAS: Total enterocyte absorption surface
TEM: Transmission electron microscopy
TER: Transepithelial resistance
TJs: Intestinal tight junctions
U: Unit of enzyme activity
